# The Dual Role of a Polyvalent IgM/IgA-Enriched Immunoglobulin Preparation in Activating and Inhibiting the Complement System

**DOI:** 10.3390/biomedicines9070817

**Published:** 2021-07-14

**Authors:** Carolin Schmidt, Sabrina Weißmüller, Fabian Bohländer, Matthias Germer, Martin König, Alexander Staus, Andrea Wartenberg-Demand, Corina C. Heinz, Jörg Schüttrumpf

**Affiliations:** 1 Department of Translational Research, Biotest AG, Landsteinerstraße 5, 63303 Dreieich, Germany; carolin.schmidt@clintricare.com (C.S.); Martin.koenig@biotest.com (M.K.); 2 Department of Analytical Development and Validation, Biotest AG, Landsteinerstraße 5, 63303 Dreieich, Germany; Fabian.bohlaender@biotest.com; 3 Preclinical Research, Biotest AG, Landsteinerstraße 5, 63303 Dreieich, Germany; Matthias.germer@biotest.com; 4 Corporate Biostatistics, Biotest AG, Landsteinerstraße 5, 63303 Dreieich, Germany; Alexander.staus@biotest.com; 5 Corporate Clinical Research & Development, Biotest AG, Landsteinerstraße 5, 63303 Dreieich, Germany; Andrea.wartenberg-demand@biotest.com; 6 Clinical Strategy & Development, Biotest AG, Landsteinerstraße 5, 63303 Dreieich, Germany; Corinacornelia.heinz@biotest.com; 7 Corporate R&D, Biotest AG, Landsteinerstraße 5, 63303 Dreieich, Germany; Joerg.schuettrumpf@biotest.com

**Keywords:** anaphylatoxins, complement factors C3, C4, CDC, IgA, IgM, IgG, immunomodulation, opsonophagocytosis, polyvalent immunoglobulin

## Abstract

Activation of the complement system is important for efficient clearance of a wide variety of pathogens via opsonophagocytosis, or by direct lysis via complement-dependent cytotoxicity (CDC). However, in severe infections dysregulation of the complement system contributes to hyperinflammation. The influence of the novel IgM/IgA-enriched immunoglobulin preparation trimodulin on the complement pathway was investigated in in vitro opsonophagocytosis, binding and CDC assays. Immunoglobulin levels before and after trimodulin treatment were placed in relation to complement assessments in humans. In vitro, trimodulin activates complement and induces opsonophagocytosis, but also interacts with opsonins C3b, C4b and anaphylatoxin C5a in a concentration-dependent manner. This was not observed for standard intravenous IgG preparation (IVIg). Accordingly, trimodulin, but not IVIg, inhibited the downstream CDC pathway and target cell lysis. If applied at a similar concentration range in healthy subjects, trimodulin treatment resulted in C3 and C4 consumption in a concentration-dependent manner, which was extended in patients with severe community-acquired pneumonia. Complement consumption is found to be dependent on underlying immunoglobulin levels, particularly IgM, pinpointing their regulative function in humans. IgM/IgA provide a balancing effect on the complement system. Trimodulin may enhance phagocytosis and opsonophagocytosis in patients with severe infections and prevent excessive pathogen lysis and release of harmful anaphylatoxins.

## 1. Introduction

The complement system is a complex network of more than 30 soluble and membrane-bound proteins, which is an essential defense mechanism against invading microorganisms [1,2]. Complement is activated via the classical pathway (CP), alternative pathway (AP) or via the mannose-binding lectin (MBL) pathway. All three pathways lead to C3b and C4b deposition onto the pathogen or target structure. Antibody and complement opsonized pathogens are cleared via opsonophagocytosis by phagocytic cells such as macrophages and neutrophils. If not phagocytosed, i.e., in the absence of phagocytes, the complement pathway may proceed, leading to the activation of the C5 convertase. This protease cleaves C5 into C5a and C5b, finally resulting in the formation of the membrane attack complex (MAC). MAC causes lysis of the pathogen via pore formation (complement-dependent cytotoxicity (CDC)). The release of anaphylatoxin C5a in turn induces the recruitment of phagocytes in order to support effective opsonophagocytosis [3].

Despite the anti-pathogen activity, this downstream complement pathway is thought to be also related to inflammation. In the case of severe infections, it may lead to excessive generation of anaphylatoxins like C3a and C5a. In addition to the aforementioned pathways, formation of anaphylatoxins may also be enhanced by the complement amplification loop, which can further augment complement activity and inflammation.

Anaphylatoxins may cause degranulation of endothelial cells, mast cells or phagocytes, which release vasoactive mediators. If the degranulation is widespread, it can cause a shock-like syndrome similar to that of an allergic reaction [4]. Furthermore, anaphylatoxins attract and activate inflammatory effector cells such as neutrophils and may cause their degranulation or release of neutrophil extracellular traps (NETs). These contain several toxic molecules directed against microbial pathogens including reactive oxygen species (ROS), myeloperoxidase, glucosidases, proteases and antibacterial peptides [5]. However, these molecules may also cause tissue damage and local inflammatory responses [6,7]. Finally, the complement system (e.g., via C5a) is able to interact with and activate the coagulation system. In the case of excessive activation, this can also lead to intravascular coagulation and thrombus formation [8,9,10].

Therefore, it is crucial that the complement system is tightly regulated by specific control mechanisms. Many surface and plasma proteins negatively regulate the CP (e.g., at the C3 convertase level) and the amplification loop (e.g., CD55 (DAF), CD35 (CR1) and CD46 (MCP), Factor I, the C4-Bindingprotein (C4-bp), and Factor H) [11,12,13,14]. 

Several studies have suggested that in addition to the regulators, the immunoglobulins (Igs) also influence and control the activity of the complement pathway in a dual manner [15,16,17]. Firstly, it is well known that specific Igs trigger complement activation by binding to target antigens, thereby facilitating binding of the first complement factor (C1q) of the classical complement pathway. Via a cascade of events, this leads to proteolysis of the complement protein C3 and the subsequent covalent attachment (deposition) of the products C3b and C4b (opsonization) onto the target. In contrast to the CP, which is mainly activated by IgM and to some extent by IgG molecules, the AP can be activated by polymeric IgA. In the latter case, C3 is either activated directly by polymeric IgA bound to the pathogen surface or recognized directly by bacterial surface molecules [18]. Polymeric IgA can also activate the MBL complement pathway [19]. MBL molecules resemble C1q and initiate downstream proteolytic steps identical to the CP.

Secondly, in several studies, it has been demonstrated that, in contrast to specific antibodies, polyvalent Ig preparations (e.g., standard intravenous IgG preparation (IVIg) or IgM-enriched preparations (e.g., Pentaglobin, containing ≈12% IgM, ≈12% IgA and ≈76% IgG)) inhibit the complement pathway non-specifically. Polyvalent IgG and IgM (but not IgA) have the ability to block activated complement factors by binding certain complement factors in a concentration-dependent manner [17,20,21,22,23,24,25,26]. Several studies described general or Fc-specific binding of polyvalent IgG to opsonins C3 and C4 [16,27,28,29,30,31,32]. Other studies demonstrated the F(ab)’2-mediated binding of IgG to anaphylatoxins C3a and C5a in the fluid phase [16,33]. It was found that specific opsonin- as well as anaphylatoxin-dependent activities are reduced by addition of IVIg preparations.

The complement inhibitory capacity of IVIgs is lower as compared to preparations with high IgM content [24,34]. In an in vitro model system, using aggregated IgG molecules coated onto a plate surface (mimicking IgG molecules bound to a pathogen surface) C3b and C4b are deposited on the aggregates upon addition of serum [24]. In contrast to IVIg, IgM-containing preparations inhibited complement deposition on aggregated IgG molecules. Similarly, in a functional CDC assay, the IgM-containing preparations also reduced complement-dependent cell cytotoxicity more effectively as compared to IVIg [23,24].

Since the complement defense mechanisms can induce inflammation and self-damage processes (e.g., in the course of severe infections, or in the presence of auto-antibodies) the dual functions of Igs provide a protective role. The Igs support phagocytosis and opsonophagocytosis and prevent excessive lysis of pathogens as well as release of inflammatory mediators. Indeed, as suggested from in vitro and in vivo experiments, Igs may protect from inflammation and self-damage [17,27,35,36,37,38,39]. However, inhibition in the assays and model systems was found to be concentration-dependent. In contrast, patients with severe infections often present a compromised immune system, i.e., reduced IgG- and IgM-plasma levels, as well as lymphocytopenia. Both IgG and IgM serum concentrations have been inversely correlated with disease severity and mortality [40,41,42]. This could in-turn imply that the opsonophagocytosis supporting and complement inhibitory functions are not sufficient and that patients may benefit from Ig supplementation therapy [43]. 

The human plasma-derived polyvalent IgM/IgA-enriched Ig preparation trimodulin (≈23% IgM, ≈21% IgA, ≈56% IgG), is currently in development for the treatment of severe infections in adults, including severe community-acquired pneumonia (sCAP) and severe coronavirus disease 2019 (COVID-19). A phase I clinical trial in healthy subjects and a phase II clinical trial in patients with sCAP, have been successfully conducted [44,45,46]. Post hoc analysis revealed a high mortality benefit in sCAP patients with a strong inflammatory response who were treated with trimodulin compared to placebo. In addition to the relevant antimicrobial activities mediated by the three antibody isotypes present in the preparation, trimodulin was shown to have immunomodulatory functions [44,47,48,49].

The aim of the present study was to characterize the activity of trimodulin regarding complement activation and inhibition. The study was performed to investigate whether trimodulin also exhibits dual functions regarding the complement system. Three questions arose from the data: (1) Is the effective, regulatory concentration of Ig observed in in vitro assays within the range of Ig concentrations in healthy humans? (2) Do Ig concentrations found in patients with severe infections suggest that inhibition of the complement system is not provided? (3) Which trimodulin dose would be required to supplement patients to provide sufficient complement modulating activity? For the first time, this in vitro activity of trimodulin was linked to effects and Ig concentrations observed in humans. The results give new insights into the complex modes of action of this unique IgM/IgA-enriched Ig preparation.

## 2. Materials and Methods

### 2.1. Immunoglobulin (Ig) Preparations 

Two Ig preparations from Biotest AG (Dreieich, Germany) were used and compared in this study: trimodulin (50 g/L), a polyvalent IgM/IgA-enriched Ig preparation, containing ≈23% IgM, ≈21% IgA and ≈56% total IgG and Intratect (50 g/L), a normal human IVIg preparation with ≥95% IgG. Formulation buffers (FB) contain 300 mM glycine in distilled water and are adjusted to pH 4.3 (trimodulin) or pH 5.0 (IVIg).

### 2.2. Opsonophagocytosis Assay (OPA) and Phagocytosis Assay (PA)

To distinguish between phagocytosis and opsonophagocytosis, either heat-inactivated or active serum was used as a source of complement. Furthermore, to investigate the role of the Igs in trimodulin, serum depleted for IgM and IgG was used (NHS^min^; normal human serum depleted of IgG and IgM, Pel-Freez, Rogers, USA), since serum depleted for IgM, IgA and IgG (Sigma-Aldrich, St. Louis, MO, USA) was found to be deficient in C1q [50].

HL60 cells (ATCC^®^ CCL240™, Manassas, VA, USA) served as effector cells in the OPA and PA and were differentiated into mature neutrophil-like granulocytes according to the protocol described by Romero-Steiner et al. [51]. Differentiation was verified by staining the HL-60 cells with an anti-CD35 and anti-CD71 antibody (BD, Franklin Lakes, NJ, USA) and analyzed by flow cytometry (FACS Canto II, BD, Franklin Lakes, NJ, USA). Differentiated and undifferentiated HL-60 cells (1.25 × 10^6^ cells in 1 mL) were seeded in a 24-well plate (Corning, New York, NY, USA) and 10 µL of AlexaFluor 488 labeled *S. aureus* bioparticle suspension (Thermo Scientific, Waltham, MA, USA) was added. Subsequently, 20 µL NHS^min^ or heat-inactivated NHS^min^ (hiNHS^min^) was added. Different dilutions of trimodulin were prepared and added to the wells with the HL-60 cells and bioparticles. The plate was incubated for 45 min at 37 °C to allow phagocytosis of the bioparticles. Following two washing steps with D-PBS, the cell pellets were resuspended in 250 µL D-PBS and 125 µL of cell suspension was transferred to each well of a 96-well V bottom plate in duplicate. For quenching of fluorescent bioparticles bound to the surface of the cells (not phagocytosed bioparticles), 30 µL of trypan blue solution (Corning, New York, NY, USA) was added. The plate was incubated for 5–10 min at 2–8 °C and washed with D-PBS. The pellet was resuspended in 100 µL D-PBS and the amount of bioparticles phagocytosed by differentiated HL-60 cells was measured by flow cytometry. For calculation of the phagocytic index (PI), the median fluorescence intensity (MedFI) of the bacteria per positive cell was multiplied with the percentage of the HL-60 cells, containing at least one bacterium (%-parent).

### 2.3. Opsonin Enzyme-Linked Immunosorbent Assay (ELISA)

The opsonin enzyme-linked immunosorbent assay (ELISA) was adjusted to the protocol described by Rieben et al. [24]. Aggregated IgG was generated by incubation of IVIg at 63 °C for 1 h and subsequent centrifugation at 3000× *g* for 10 min to remove insoluble aggregates. A 96-well flat bottom plate (Nunc MaxiSorp, Invitrogen, Carlsbad, CA, USA) was coated with 100 µL, 0.1 µg/mL aggregated IgG in 0.05 M carbonate bicarbonate buffer (pH 9.4; Sigma-Aldrich, St. Louis, MO, USA) overnight at 2–8 °C. The following day, the coating solution was discarded and the plate was washed three times with 300 µL/well standard Tris-buffered saline with Tween 20 (TBS-T) washing buffer. The following steps were performed on wet ice. EDTA-plasma (prepared in-house) was diluted 100-fold in Gelatin Veronal Buffer with Mg^2+^ and Ca^2+^ containing 0.05% Tween 20 (GVB^++^; Boston BioProducts, Ashland, MA, USA). Various dilutions of trimodulin and IVIg in the corresponding FB were prepared and 50 µL of these dilutions were mixed with 50 µL EDTA-plasma on the aggregate-coated plates. As negative controls, trimodulin or IVIg were mixed with 50 µL GVB^++^ (no complement). As positive control, 50 µL EDTA-plasma was mixed with 50 µL GVB^++^ (full complement activation). The plate was incubated for 1 h at 37 °C and subsequently washed three times with 300 µL/well TBS-T washing buffer. For detection of C3b and C4b deposition on the aggregates, a rabbit anti-human C3b or C4b antibody that is also capable of detecting C3c/C4c as well as iC3b/iC4b (Dako Agilent, Santa Clara, CA, USA), a HRP-labelled goat anti-rabbit antibody conjugate (Dako Agilent, Santa Clara, CA, USA) and tetramethylbenzidine substrate were used. After stopping the reaction, the absorbance was measured in a spectralphotometer (Tecan, Männedorf, Switzerland) at 450/690 nm.

### 2.4. Anaphylatoxin C5a ELISA

A 96-well flat bottom plate was coated with 100 μL, 20 μg/mL zymosan, a carbohydrate complex derived from yeast cell walls (Sigma-Aldrich, St. Louis, MO, USA), in carbonate bicarbonate buffer and incubated overnight at 2–8 °C (plate 1). Simultaneously, plate 2 was either coated with 5 μg/mL goat anti-human IgG+IgA+IgM antibody mix (for trimodulin immobilization; Abcam, Cambridge, UK) or with 5 μg/mL goat anti-human IgG anti-body (for IVIg immobilization; Abcam Cambridge, UK) and incubated overnight at 2–8 °C as well. Then, the plates were washed three times with 300 μL TBS-T washing buffer. EDTA-plasma was diluted 50-fold in GVB^++^ and 60 μL of this dilution was added to the wells of plate 1 and the plate was incubated at 37 °C. The zymosan coating activates the AP antibody independently and induces the generation of anaphylatoxins in human EDTA-plasma in the first plate. After 1 h, 50 μL of the supernatant (containing the activated complement factors, including C5a) was transferred to plate 2. Since C5a, compared to C3 and C4, is only present in serum at very low concentrations in the range of 3.9–250 ng/mL [52], trimodulin and IVIg were pre-diluted 1250-fold. Then, a 1.8-fold serial dilution in the corresponding FB was prepared. We distributed 50 μL of the dilutions and controls to plate 2 and mixed. By incubating the plate for 1 h at 37 °C, trimodulin as well as IVIg were Fc-immobilized onto plate 2 by the antibody mix coating, but still able to Fab-bind the activated anaphylatoxins. The supernatant with the remaining unbound C5a molecules was then transferred to an anti-human C5a coated plate 3 (BD, Franklin Lakes, NJ, USA). C5a was detected according to the manufacturers’ instruction in a spectralphoto-meter (Tecan, Männedorf, Switzerland) at 450/690 nm.

### 2.5. Complement-Dependent Cytotoxicity (CDC) Assay

A CDC assay was optimized showing high lysis rates of the target cells. Ramos (RA1) (ATCC^®^ CRL-1596™, Manassas, VA, USA) cells, a B lymphocytic cell line that expresses the surface antigen CD20, were washed twice and adjusted to 5 × 10^6^ cells/mL in CDC assay buffer (serum-free RPMI-1640 medium with 10 mM HEPES). We distributed 50 µL of the cells in a V-bottom 96-well plate (Greiner Bio-One, Frickenhausen, Germany) and stored at 37 °C and 5% CO_2_ (plate 1). Rituximab (MabThera, Roche, Basel, Switzerland) was pre-diluted to 1600 µg/mL in CDC assay buffer and 10-fold serial dilutions were prepared in plate 2. Additionally, 150 µL of 160 µg/mL Rituximab in CDC assay buffer was distributed on the plate. For the two-fold serial dilutions of trimodulin and the IVIg control, their appropriate FB was used. In plate 3, 30 µL of 80% normal human serum (NHS) in CDC assay buffer was distributed. Then, 15 µL of the diluted Ig preparations of plate 2 was mixed with 30 µL of 80% NHS on plate 3 and incubated for 1 h at 37 °C and 5% CO_2_. Following this, 15 µL of 160 µg/mL Rituximab was added to the wells containing the pre-incubated Ig preparations and NHS. Subsequently, 50 µL/well of these dilutions was transferred to the prepared Ramos (RA1) cells (on plate 1) and incubated for 65 min at 37 °C and 5% CO_2_. We added 10 µL of 8% Triton-X 100 (Sigma-Aldrich, St. Louis, MO, USA) to the wells containing Ramos (RA1) cells in assay buffer only, which served as 100%-killing control and was incubated for additional 15 min at 37 °C and 5% CO_2_. To monitor the CD20 expression and to distinguish living from dead cells, the Ramos (RA1) cells were stained with a mouse anti-human CD20-APC conjugated antibody (BD, Franklin Lakes, NJ, USA) as well as with propidium iodide (Invitrogen, Carlsbad, CA, USA), according to the manufacturers’ instruction. After incubation, the plates were measured by flow cytometry (FACS Canto II, BD, Franklin Lakes, NJ, USA) and the percentage of dead cells [%-parent] detected indicated CDC (lysis). The lysis of untreated cells (without Rituximab) was set to 0% lysis and thus 100% cell viability.

### 2.6. SDS-PAGE and Western Blot Analysis

Supernatants of the opsonin ELISA were denatured (10 min at 70 °C) and separated on an 8% bis-tris gel (NuPage system, Thermo Scientific, Waltham, USA). After electrophoresis (200 V, 45 min), the gel was blotted onto a nitrocellulose membrane (Thermo Scientific, Waltham, MA, USA) according to the standard online protocol of Thermo Scientific (Waltham, MA, USA) for wet blots with fluorescent detection [53]. For C3b detection, a rabbit anti-human C3c antibody (that is also able to detect C3b as well as iC3b; Agilent, Santa Clara, CA, USA) and a donkey anti-rabbit antibody (Biotium, San Francisco, CA, USA) were used. The membrane was scanned and C3b was detected with a Typhoon Imager (GE Healthcare, Chicago, IL, USA).

### 2.7. Phase I Trial Design

The phase I trial (EudraCT 2007-005855-41) with trimodulin (BT086, 5% solution for intravenous use, Biotest AG, Dreieich, Germany) was a prospective, randomized, placebo-controlled, single-blind, first-in-man clinical phase I trial conducted 2009. This prospective randomized, placebo-controlled, single-blind trial was conducted in a single center in Germany in 24 healthy subjects to investigate safety, tolerability and the PK/PD properties of trimodulin. The trial was conducted in accordance with the International Council for Harmonisation, Good Clinical Practice standards, and the Declaration of Helsinki and with local institutional review board/independent ethics committee approval. All subjects provided written informed consent. Adult healthy subjects, aged 18 years and above, were enrolled and randomized to receive one of the three different single doses (94.2 mg/kg, 182.6 mg/kg, 273.9 mg/kg) of trimodulin (*n* = 5 per dose group) or placebo (*n* = 3, 1 subject per dose group, 1% albumin solution), or multiple dosages of 182.6 mg/kg, repeated on five consecutive days (*n* = 6). Infusions were started at a rate of 0.1 mL/min, increasing by 0.1 mL every 10 min to a maximum of 0.5 mL/min (target infusion rate). Subjects were followed up until day 22 (single-dose group) or 29 days (multiple-dose group). Pharmaco-kinetic profiling of IgM following trimodulin (BT086) treatment was the primary objective of this trial. 

### 2.8. Complement Assessments in Healthy Subjects

In the phase I trial, the C3 and C4 serum concentrations were determined in subjects infused with single dosages of trimodulin at screening, day 1 (pre infusion), day 1 (post infusion), day 3, 5, 9 and 22. In subjects infused with multiple dosages of trimodulin, serum C3 and C4 concentrations were determined at screening day 1 (pre and post), 2 (pre and post), 3 pre, 4 pre, 5 pre, 6, 8, 12 and 29. In a post hoc assessment, the %-change from baseline in C3 and C4 complement serum concentrations was assessed. For single-dosed subjects baseline (day 0) was defined as the mean of the values obtained at screening and day 1 (pre). For multiple-dosed subjects, baseline represented the screening value. The %-change in complement level before and after treatment was calculated in each subject by comparing baseline (day 0) to levels at post infusion days (%-change = ((Value Day X/baseline) − 1) × 100%).

### 2.9. Complement Assessments in Severe Community-Acquired Pneumonia (sCAP) Patients

A randomized, double-blind, placebo-controlled phase II trial was conducted with trimodulin (BT086) in adult sCAP patients on mechanical ventilation. Patients were enrolled in Germany, Spain, Belgium and England 2011–2015 (EudraCT 2010-022380-35 and NCT01420744). The C3 and C4 serum concentrations were determined only in three patients with sCAP requiring invasive mechanical ventilation (see [44] for trial design). The patients were infused with multiple dosages of trimodulin (182.6 mg/kg) for five consecutive days. Serum C3 and C4 concentrations were determined at screening (pretreatment), day 2, 4 and 14. The %-change from baseline in serum C3 and C4 concentrations was assessed as above by comparing baseline (day 0) to levels on post-infusion days (%-change = ((Value Day X/baseline) − 1) × 100%).

### 2.10. Statistical Analyses

In this exploratory study, descriptive statistical analyses were performed using GraphPad Prism Software (Version 6.07). Pairwise comparisons between the various trimodulin concentrations and untreated controls were made using unpaired Wilcoxon–Mann–Whitney tests.

## 3. Results

### 3.1. Trimodulin Induces Phagocytosis and Opsonophagocytosis in a Concentration-Dependent Manner

To investigate the complement activating and inhibiting functions of trimodulin, initially phagocytosis and opsonophagocytosis supporting functions of trimodulin were analyzed using a modified PA/OPA assay. 

**Figure 1:**
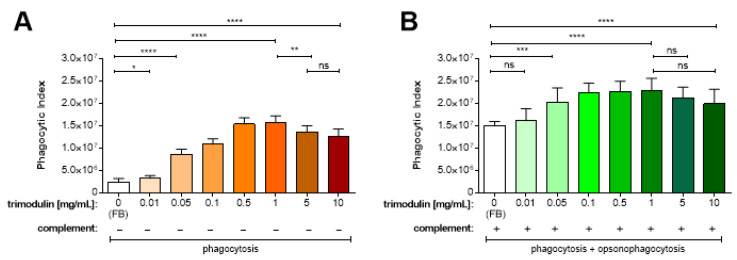
Trimodulin induces concentration-dependent phagocytosis and opsonophagocytosis of *S. aureus* bioparticles. (**A**) Phagocytosis and (**B**) (opsono)phagocytosis of *S. aureus* bioparticles by differentiated HL-60 cells in the absence of complement (−, with hiNHS^min^), or in the presence of active complement (+, with NHS^min^), respectively. Additionally, assays included no (0, white bars, with FB only) or different trimodulin concentrations as indicated below the figures, representing final trimodulin concentrations in the assays. Bars represent mean + standard deviation (SD, *n* = 6 independent experiments). Reactions without active complement (−) display phagocytosis activity. Reactions with active complement (+) display phagocytosis plus opsonophagocytosis. Statistical analyses were performed using an unpaired Wilcoxon–Mann–Whitney test. Significance is shown with asterisk indicating a *p*-value of: * *p* < 0.05; ** *p* < 0.01; *** *p* < 0.0005; **** *p* < 0.0001; ns: non-significant. FB: formulation buffer; NHS^min^: normal human serum depleted for IgM and IgG; hiNHS^min^: heat-inactivated NHS^min^; SD: standard deviation.

Phagocytosis (PA) is shown by incubation of differentiated HL-60 cells and *S. aureus* bioparticles in the presence of heat-inactivated serum (hiNHS^min^, inactive complement, containing only IgA). Using FB (0 mg/mL trimodulin), a PI of about 2.4 × 10^6^ was detected, representing baseline phagocytosis activity of HL-60 cells (Figure 1A, white bar). Compared to this, the PI already significantly increased to 3.2 × 10^6^, if 0.01 mg/mL trimodulin was added (Figure 1A, *p* = 0.0433). This activity of the HL-60 cells further increased dose-dependently to 1.6 × 10^7^ at 1 mg/mL trimodulin (*p* < 0.0001). This shows that trimodulin induces complement-independent phagocytosis of the bioparticles. Above 1 mg/mL a significant drop in PI was observed (e.g., *p* = 0.0089 compared to 5 mg/mL) in this setting. Nevertheless, at 10 mg/mL phagocytosis was still significantly (*p* < 0.0001) increased as compared to the FB control. In the absence of active complement, the phagocytosis-stimulating activity of trimodulin is mostly IgG- and IgA-dependent, since the HL-60 cells exhibit both Fc-γ receptors (FcγR) as well as Fc-α receptors (FcαR) on their surface [48,54,55]. The IgM-binding Fc-μ receptor (FcμR) is not expressed. 

Opsonophagocytosis (OPA) was observed if NHS^min^ (with active complement) was applied to the differentiated HL-60 cells. This allows binding of complement to the *S. aureus* bioparticles, which is most efficiently induced by IgM molecules in the presence of active serum. However, in active complement without trimodulin (FB control), the HL-60 cells showed a PI of about 1.5 × 10^7^. Thus, in the absence of IgM and IgG antibodies, a marked increase in phagocytosis and opsonophagocytosis of the *S. aureus* bioparticles occurs (Figure 1B, white bar). This baseline activity was mediated on the one hand by binding of IgA (present in NHS^min^) to the pathogens inducing phagocytosis and on the other hand via direct activation and IgA-mediated activation of the alternative and MBL complement pathways (inducing opsonophagocytosis) on the bioparticles. Nevertheless, baseline opsonophagocytosis was significantly (up to *p* < 0.0001) increased by the addition of trimodulin (Figure 1B, green bars). The opsonophagocytosis of the bioparticles increased significantly compared to FB after the addition of 0.05 up to 1 mg/mL trimodulin (*p* = 0.002 up to *p* < 0.0001), reaching a peak and plateau of 2.0 to 2.3 × 10^7^. 

These results show that the combination of trimodulin and complement improved the phagocytic and opsonophagocytic activity of HL-60 cells.

### 3.2. Trimodulin Reduces the Detection of Opsonins C3b and C4b in a Concentration-Dependent Manner

**Figure 2:**
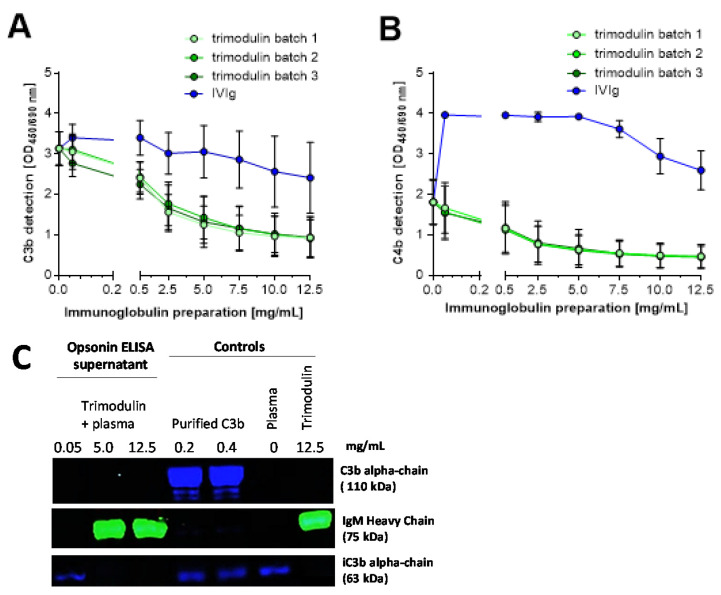
Trimodulin inhibits the detection of the opsonins C3b and C4b concentration-dependently. Opsonin binding assay (ELISA) showing different dilutions of three batches trimodulin (green) and a single intravenous IgG preparation (IVIg) batch (blue). Deposition of (**A**) C3b and (**B**) C4b was detected with an anti-human C3b or C4b antibody, respectively. Data points represent the mean from six independent experiments performed in duplicates. Error bars indicate the standard deviation (±SD). (**C**) Western blot analysis of the supernatant of the opsonin ELISA. Loading of the different controls and trimodulin samples on the gel is indicated. C3 fragments (C3b, iC3b, C3c) were detected with a goat anti-human C3c antibody (Dako Agilent, Santa Clara, USA) and a secondary CF488A-labeled donkey anti-goat antibody (blue; Biotium, San Francisco, CA, USA). IgM was detected with a mouse anti-human IgM antibody and a CF588-labeled donkey anti-mouse antibody (green). The plasma control contains EDTA-plasma only, while the trimodulin control contains trimodulin without EDTA-plasma. OD: optical density; ELISA: enzyme-linked immunosorbent assay.

The results presented in Figure 1 indicate that the level of phagocytosis and opsono-phagocytosis of *S. aureus* bioparticles by HL-60 cells is dependent on trimodulin concentration. This result suggests that trimodulin supports these immunological clearance mechanisms efficiently in the presence of complement. However, previous data have shown that IgM and to a lesser extend IgG can interact with C3b and C4b resulting in the inhibition of their functions [17,22]. Based on the observed data at the investigated concentrations, such potential inhibition with trimodulin did not seem to affect opsonophago-cytosis processes. Therefore, these interactions of Igs with opsonins were investigated for trimodulin to verify previous results. Since trimodulin has a unique Ig composition and is produced from a distinctive and unique production process, binding activity might be different compared to standard IVIg. The concentration range required for interactions, the extent of interactions, and the role of IgM/IgA were investigated in comparison to a standard IVIg using C3b- and C4b-specific ELISA assays (Figure 2A,B).

By adding increasing concentrations of trimodulin, a decrease in C3b detection was observed, with highly comparable values using three batches (Figure 2A). In comparison, the lowest IVIg concentration of 0.05 mg/mL initially caused a slight increase in C3b detection. Thereafter, the detection of C3b dropped slowly with increasing IVIg concentration. At the highest Ig concentration (12.5 mg/mL), C3b detection decreased by ≈73% (from OD_450/690_ ≈ 3.3 to ≈0.9) with trimodulin and ≈27% (from OD_450/690_ ≈ 3.3 to ≈2.4) with IVIg.

Similar results were obtained for C4b (Figure 2B). After the addition of trimodulin, the detection of C4b on aggregated IgG was reduced in a concentration-dependent manner and the results of the herein used three batches were highly comparable. At the highest trimodulin concentration (12.5 mg/mL), C4b detection decreased ≈74% (from OD_450/690_ ≈ 1.8 to ≈0.4). Remarkably, the addition of 0.05 mg/mL IVIg caused a marked initial increase in C4b detection. The reason for this specific and reproducible C4b deposition induced by IVIg is currently unknown, but is obviously related to the differences in Ig composition of the different preparations. Subsequent C4b detection remained largely unchanged up to a concentration of 5 mg/mL IVIg and then slightly dropped.

It was postulated that the interaction of IgG and IgM with the complement factors C3b and C4b occurs via “scavenging in the fluid phase” meaning that C3b/C4b deposited onto the tissue/pathogen is displaced by preferentially binding to Igs in the fluid phase [21,23,24,56]. This would prevent excessive pathogen and host-tissue lysis. Most of the C3b and iC3b generated during complement activation binds to the surface through a covalent bond. These bonds may be unstable, forming fluid phase C3b. Surface-bound C3b and iC3b as well as its breakdown product C3d are recognized by several receptors on lymphoid and phagocytic cells, culminating in phagocytosis, if linked to the target. However, we investigated this hypothesis and the results of the supernatant (fluid phase) analysis showed that C3b was undetectable in the fluid phase with increasing trimodulin concentration (Figure 2C). The plasma control showed no C3b detection after 1 h of assay time. This observation could be attributed to the very short half-life of C3b. At the end of its half-life, C3b is cleaved into iC3b, which was detected accordingly. Nevertheless, iC3b did not increase in the fluid phase with increasing trimodulin concentrations. This would argue for a decrease in C3b/iC3b detection, e.g., possibly due to attaching and “covering” on the surface, rather than scavenging/displacing of deposited C3b/iC3b by trimodulin in the fluid phase. In line with this finding is the large pentameric form of IgM (with a molecular weight of 990 kDa), which is possibly more effective in masking pathway-essential binding sites of interacting complement factors as compared to the smaller IgG monomers (150 kDa).

In conclusion, both opsonin ELISAs showed that trimodulin has the ability to interact with activated complement factors, as detection of C3b and C4b was reduced in this in vitro setting. This reduction in the detection of C3b and C4b is likely IgM- and to a lesser extent IgG-dependent and clearly differentiates these two preparations in their activity.

### 3.3. Trimodulin Reduces CDC Lysis

The results presented in Figure 2 indicate that trimodulin interacts in a different way with different activated complement factors compared to IVIg. At the investigated concentrations, the IgM/IgA components seem to bind to C3b and C4b more efficiently than IgG alone. Therefore, it was investigated whether this binding activity of IgM/IgA in trimodulin also results in blocking the activity of the terminal complement pathway. The impact of this interaction on the downstream lytic complement pathway, leading to cell lysis via CDC, was analyzed.

A flow-cytometer based CDC assay was developed using the CD20-specific antibody Rituximab and Ramos (RA1) cells as target. Rituximab is able to induce CDC-lysis on these CD20-positive cells [57]. The CDC assay was optimized to show high baseline lysis rates of the Ramos (RA1) cells by Rituximab and to show relevant inhibition by trimodulin (data not shown). Various control experiments were performed to assess the assay specificity. Trimodulin was found not to be able to directly interact with the chimeric antibody Rituximab and is not able to specifically interact with CD20 on the Ramos (RA1) cells (data not shown). The detection of CD20 on the Ramos (RA1) cells before as well as after trimodulin addition was high (>95%), showing that unspecific overlay by antibodies in trimodulin did not occur. Moreover, an additional control experiment showed that trimo-dulin was not able to induce the CDC-lysis of Ramos cells in the absence of Rituximab (data not shown).

**Figure 3:**
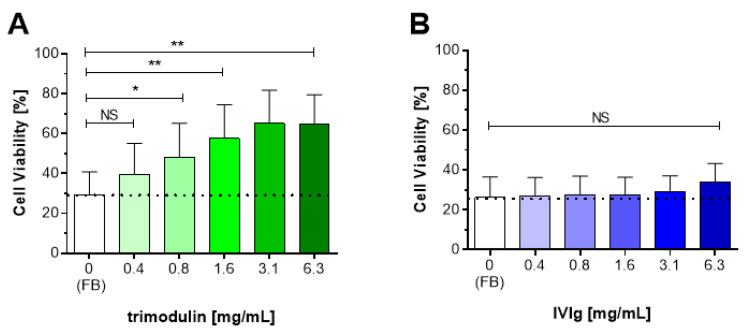
Trimodulin reduces complement-dependent cytotoxicity (CDC) lysis activity in a concentration-dependent manner. Percentage of viable Ramos (RA1) cells after pre-incubation in normal human serum (NHS) with Rituximab (20 µg/mL) plus increasing concentrations of (**A**) trimodulin or (**B**) IVIg inducing CDC lysis via the classical pathway (CP). Cell viability of Ramos cells was analyzed by staining the membrane attack complex (MAC)-perforated cells with propidium iodide and measurement in a flow cytometer. As control, the FB plus Rituximab (0 mg/mL Ig) was used to determine the base level of CDC (white bars). Bars and error bars indicate mean + SD from six independent experiments performed in duplicates. Statistical analyses were performed using an unpaired Wilcoxon–Mann–Whitney test. Significance is shown ns: non-significant or with asterisks indicating a *p*-value: * *p* < 0.05; ** *p* < 0.01. FB: formulation buffer; CP: classical pathway; SD: standard deviation.

After optimization and specificity testing, it was investigated whether the interaction of trimodulin with C3b and C4b ultimately affects the lytic pathway of the complement system and thus inhibits lysis of the Ramos (RA1) cells by Rituximab (Figure 3A). After addition of Rituximab alone (FB; untreated control), approx. 30% of the Ramos (RA1) cells survived, i.e., 70% of the cells were lysed by CDC. Compared to this, cell survival increased significantly (up to *p* = 0.0043) and concentration-dependently after addition of trimodulin. A plateau was reached resulting in approximately 65% survival after the addition of 3.1–6.3 mg/mL trimodulin. 

Addition of IVIg (Figure 3B) showed that the mean cell survival increased non-significantly (*p* = 0.2403) from 26% (FB) to 34% after addition of the highest IVIg concentration (6.3 mg/mL).

Thus, trimodulin inhibited complement-dependent Ramos (RA1) cell lysis concentration-dependently and more effectively as compared to IVIg. The increase in Ramos (RA1) cell viability and consequently the decrease in complement-dependent lysis suggests that this is indeed due to a direct complement inhibition by trimodulin. This result supports the data shown above indicating the concentration-dependent interaction of IgM/IgA with C3b and C4b (Figure 2).

### 3.4. Trimodulin Reduces the Detection of Anaphylatoxin C5a in a Concentration-Dependent Manner

Anaphylatoxins are factors released during the formation of the MAC complex, which leads to cell lysis via CDC. Primarily, the release of these anaphylatoxins induces recruitment of phagocytes to the site of infection. However, C5a is also a key inflammatory anaphylatoxin generated by the classical, lectin and alternative complement pathways. Previous data have shown that IgG can interact with the anaphylatoxins C5a and C3a possibly inhibiting their inflammatory properties [33]. At concentrations of 3–5 mg/mL first inhibitory activity was detected in a CDC-like assay measuring Ca2+ influx in HMC1 cells. Regarding the interaction of anaphylatoxins with IgM and/or IgA nothing is well-known.

**Figure 4:**
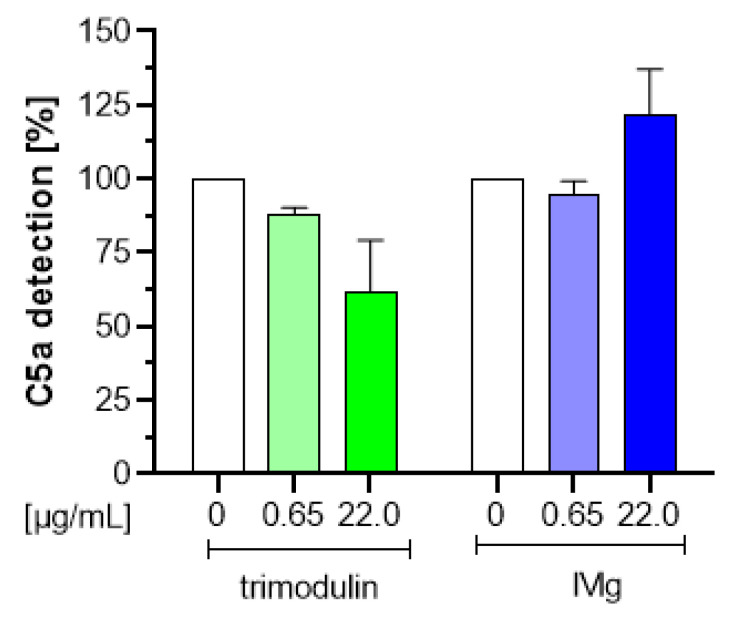
Trimodulin reduces C5a detection in a concentration-dependent manner. Anaphylatoxin binding assay showing different dilutions of trimodulin (green) and IVIg (blue) compared to the formulation buffer control (0, white bars). C5a was generated with zymosan via the AP and its binding to Igs was analyzed. Free C5a was detected by using a commercial C5a ELISA Kit. The amount of unbound C5a was set to 100% in the FB control. Data represent mean + SD from three independent experiments performed in duplicates. AP: alternative complement pathway; FB: formulation buffer; SD: standard deviation; ELISA: enzyme-linked immunosorbent assay.

To investigate whether IgG in trimodulin also interacts with anaphylatoxin C5a, a three-step anaphylatoxin ELISA was performed. Zymosan was used to generate C5a in vitro and the capturing activity of trimodulin and IVIg was investigated by analyzing residual C5a in the supernatant in the three-step ELISA. As shown in Figure 4, a slight decrease in the generated amount of C5a (≈38%; Figure 4, green) was only observed with trimodulin, which was dependent on the trimodulin concentration. 

To investigate, if this was solely an IgG-dependent effect, the decrease in C5a detection was compared using IVIg in the same concentration range. No reducing effects on the generated amount of C5a detection was observed at the investigated IVIg concentrations; Figure 4, blue). These preliminary results suggest that IgM and/or IgA in trimodulin is able to interact with the anaphylatoxin C5a even at low concentrations.

### 3.5. The Ig Concentration Range Used In Vitro Matches with Ig Levels In Vivo

The in vitro data described above show that trimodulin enhances phagocytosis and opsonophagocytosis, reduces the detection of C3b, C4b and C5a and inhibits CDC lysis in a concentration-dependent manner. To answer the question, if such functions are relevant in humans e.g., patients with infections, the Ig concentrations used in in vitro assays were compared to a detailed analysis of Ig concentrations in healthy subjects and sCAP patients before and after treatment with trimodulin. These concentrations are given for each Ig class in Table 1.

As indicated in Table 1, the highest IgA and IgG concentrations in the assays were in the range of untreated healthy subjects and sCAP patients. Post-trimodulin treatment, the IgA and IgG concentrations were approximately 1.5- to 2-fold higher and similar to those observed in the in vitro assays. After five trimodulin doses, serum IgA and IgG concentrations in healthy human subjects and sCAP patients were raised to the upper and middle reference range respectively when compared to the normal reference range.

The highest IgM concentrations used in the assays (2.9 mg/mL) are reached in the untreated healthy subjects, but not in the sCAP patients. Baseline IgM in healthy subjects is higher than in patients, where IgM consumption is most likely lower. Here, five dosages of trimodulin resulted in IgM plasma concentrations above the reference range and in the upper concentration range of the in vitro assays. In sCAP patients, the IgM concentration at baseline is lower and IgM consumption via opsonophagocytosis is most likely higher due to infection. The observed IgM concentrations in patients suggest that inhibition of complement terminal lytic pathway is reduced in the patients. The highest IgM concentrations used in the CDC assays (1.45 mg/mL) are within the IgM concentration reached in the patients after trimodulin treatment on five consecutive days. The IgM concentration reached the upper range of the normal reference range in the sCAP patients.

Thus, the first two questions ((1) Is the effective, regulatory concentration of Ig observed in in vitro assays within the range of Ig concentrations in healthy humans? (2) Do Ig concentrations found in patients with severe infections suggest that inhibition of the complement system is not provided?) have been addressed and, subsequently, affirmed.

### 3.6. Trimodulin Induces Complement Consumption in a Concentration-Dependent Manner in Human Subjects

**Figure 5:**
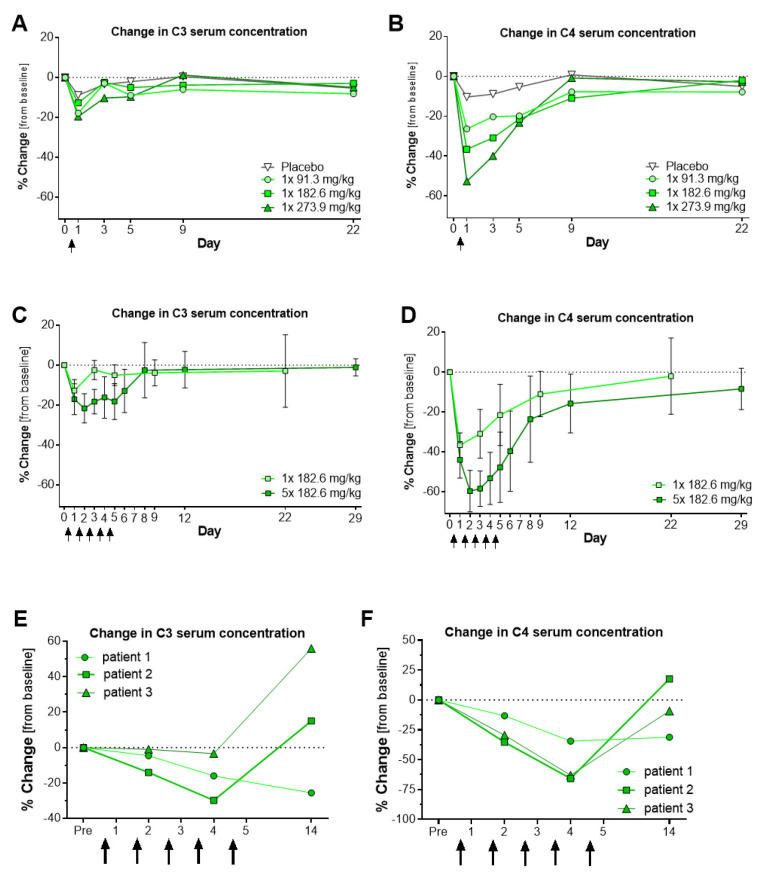
Pharmacodynamics of complement factor C3 and C4 upon trimodulin treatment in healthy subjects and sCAP patients. (**A**–**D**) Results from the phase I clinical trial with trimodulin (Study 970) showing the change in C3 and C4 serum concentrations before (Day 0), during and after single (1x 182.6 mg/kg BW, (**A**,**B**), *n* = 4) or multiple (5x 182.6 mg/kg BW, (**C**,**D**), *n* = 6) doses of trimodulin (arrows) in healthy subjects. Multiple doses were given on five consecutive days 1 through 5. (**E**,**F**) Results from the phase II clinical trial with trimodulin (Study 982, CIGMA). Multiple (5x 182.6 mg/kg BW) doses of trimodulin (arrows) were given to three patients with sCAP. Complement was assed at the end of infusion. Change was calculated as percentage change (% change) from baseline per subject and the mean was calculated per treatment group. ((**A**,**B**): mean values, (**C**,**D**): mean values ± SD with *n* = 6; (**E**,**F**): single patient values). BW: body weight; SD: standard deviation; C3: complement factor 3.

The experiments shown above revealed that trimodulin supports phagocytosis and opsonophagocytosis of pathogens (Figure 1). In human plasma, this would result in C3 and C4 consumption due to clearance. This effect was analyzed in healthy human subjects (Study 970). First, single dosages of trimodulin were given at different concentrations to healthy human subjects and C3 as well as C4 serum levels were monitored (Figure 5A,B). Due to the supplementation of antibodies, a brief concentration-dependent decrease in the C3 and C4 levels was observed on the first day of treatment. The complement levels returned to baseline levels during the following 3 to 9 days. This result shows that complement levels decrease dose-dependently during provision of a new antibody repertoire from >1000 different donors to the healthy subjects. It implies that higher trimodulin doses induced more phagocytosis and opsonophagocytosis of pathogens, common bacteria or, for example, natural IgM-mediated house-keeping clearance in the host. 

Since the in vitro experiments showed that phagocytosis is dependent on trimodulin concentration, a higher dosage should accordingly decrease C3 and C4 plasma levels further. The subjects were treated on five consecutive days (total dose: 913 mg/kg), gradually increasing the Ig serum concentration. Figure 5C,D shows that plasma C3 and C4 concentrations decreased until day 2 after the second infusion, suggesting additional consumption up to day 2. On day 3, C3 and C4 concentrations did not decrease further, despite increasing the trimodulin dose. On day 4 and 5, the C3 and C4 concentrations gradually increased again, despite additional trimodulin treatments. It seems that at these concentrations, the consumption is reduced and point to this dual effect. They are in agreement with the in vitro phagocytosis data presented in Figure 1A,B. At high trimodulin concentrations above 5–10 mg/mL (>2.8 mg/mL IgG) significant inhibition in phagocytosis occurred as compared to lower concentrations. However, phagocytosis was still increased as compared to the FB control. In the healthy subjects, this IgG concentration range is already reached before treatment (10.4 mg/mL IgG, *n* = 24). For the six subjects treated with five doses of trimodulin baseline IgG was 9.0 mg/mL, which increased on day 1 after the end of infusion to a concentration of 10.2 mg/mL, to 12.1 mg/mL on day 3 pre-infusion. On day 5, a serum IgG concentration of 14.6 mg/mL IgG was reached. 

Regarding the opsonophagocytosis activity stimulated by IgM, the mean baseline IgM concentrations in the healthy subjects was 1.1 mg/mL. Pharmacokinetic analysis of IgM concentrations revealed that in the healthy subjects at the end of trimodulin infusion on day 2, a concentration of 2.1 mg/mL IgM was reached, 2.5 mg/mL on day 3 (not shown) and 2.9 mg/mL on day 5 (Table 1). In vitro, a concentration of 5–10 mg/mL trimodulin slightly reduced opsonophagocytosis (Figure 1B), but requires a higher dose of trimodulin >10 mg/mL (>2.3 mg/mL IgM) to significantly inhibit. This concentration was reached after day 2 in the healthy subjects, but not in the sCAP patients (Table 1). It would therefore be of interest, to investigate C3 and C4 consumption in patients with sCAP, who are expected to have lower IgM and IgG baselines. A preliminary analysis was performed with C3 and C4 data from three sCAP patients (Figure 5E,F). Complement concentrations were assessed on day 2, day 4 during the treatment phase and on day 14 (9 days after the last trimodulin infusion). The results showed that complement consumption is detectable at least until day 4 and this supports that trimodulin improves clearance in the patients by phagocytosis and opsonophagocytosis. On day 4, an IgM concentration of 1.9 mg/mL was reached in the 10 patients analyzed in the PK set (Table 1).

Thus, in answer to the third question ((3) Which trimodulin dose would be required to supplement patients sufficiently to provide complement modulating activity?) the data suggest that at least four doses in sCAP patients further enhanced C3 and C4 consumption. This indicates enhanced clearance via phagocytosis and opsonophagocytosis, whereas CDC lysis was inhibited at the Ig concentration ranges reached and warrants investigation after five dosages of trimodulin in future trials.

## 4. Discussion

**Figure 6:**
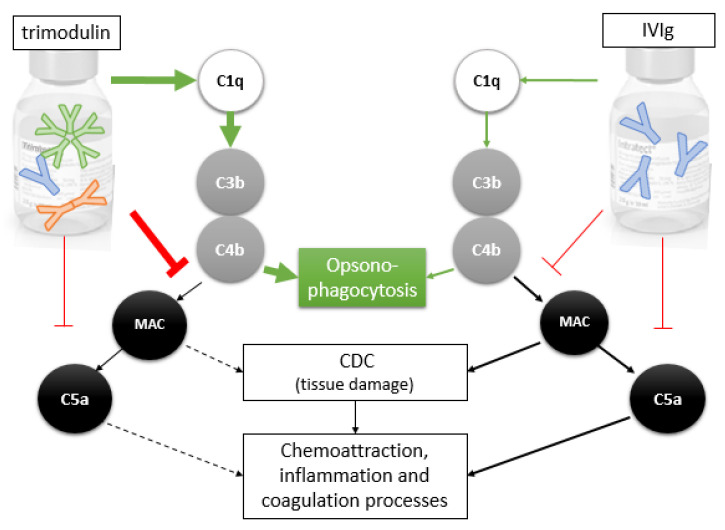
Interactions of trimodulin and IVIg with different complement factors in the classical complement pathway. Due to IgM content trimodulin activates the classical complement pathway effectively (bold green arrows) compared to IVIg (thin green arrows). This leads to effective C3b/C4b opsonization of pathogens, opsonophagocytosis and to pathogen clearance. Furthermore, trimodulin inhibits the downstream CDC pathway by reducing the formation of the MAC complex (bold red blocking line) more effectively as compared to IVIg (thin red blocking line) and consequently the release of toxic mediators released by pathogens, or in case of autoimmune diseases by the host cells. The release of toxins results in inflammation, as well as the recruitment of neutrophils, which secrete more inflammatory cytokines. Both inflammation and C5a release induce coagulation processes.

The data presented in this study indicate that the IgM/IgA-enriched Ig preparation trimodulin exhibits a dual role on the complement system by balancing activation as well as inhibition via interactions with different complement factors (Figure 6). Here, we have shown for the first time that trimodulin regulates pathogen clearance and complement activity. Trimodulin regulation includes the concentration-dependent modulation of phagocytosis and opsonophagocytosis activity of neutrophil-like cells, as well as the concentration-dependent modulation of lytic activity of the complement. In this way, trimodulin may offer efficient protection against invading pathogens through the initiation of phagocytosis, opsonophagocytosis and lysis (anti-pathogen mode of action), but simultaneously prevents or protects against over-activation of the complement system (anti-inflammatory mode of action).

Our results are in agreement with previous studies undertaken in different models, but also provide new insights. Regarding phagocytosis, increasing trimodulin concentrations enhanced phagocytosis and opsonophagocytosis (Figure 1). This is one of the most important anti-pathogenic mechanisms as it clears immune complexes in a host-protective manner without releasing inflammatory byproducts. This process, however, is inhibited at too high Ig concentrations (e.g., 20 mg/mL trimodulin, data not shown). This could be an assay-related effect due to FcR saturation of the neutrophils or via upregulation of inhibitory receptors on the HL-60 cells [48,59,60], but may also occur in vivo as suggested in Figure 5A–D in the absence of infection. It is possible that the rebound of C3 and C4 in the serum of the healthy subjects, after three of five consecutive dosages of trimodulin, was due to such saturation (Figure 5C,D). In patients with severe infections, the baseline IgM and IgG concentrations are regularly lower than in healthy subjects, possibly not reaching such saturation as Figure 5E,F suggest. Trimodulin treatment in the patients suggests that the Ig concentrations reached after five treatments are sufficient to support effective phagocytosis (IgA, IgG) and opsonophagocytosis (IgM) and that sufficient inhibition of the lytic pathway (IgM) is provided. Trimodulin may well prevent too high concentrations of C5a being released at and that thus C5a-induced processes like inflammation, coagulation, platelet activation, leukocyte recruitment and endothelial cell activation are reduced. Such processes have been found to play a pathological role in severe infections including COVID-19.

In addition to FcR saturation, it was discussed that opsonophagocytosis may also be reduced at extremely high IgG concentrations (50 mg/mL) by binding of IgG to the opsonins C3b as well as C4b molecules extending these deposited factors from the surfact into the fluid phase. This would prevent the complement deposition or reduce opsonization on target cells [15,16,26]. Rieben et al. and Roos et al. reported that IgM- as well as IgM/IgA-enriched preparations (Pentaglobin and IVIgM with 70% IgM) were more efficient as IVIg at a dose range of 0.03 to up to 25 mg/mL [23,24]. The IgM preparations efficiently inhibited the deposition of C3, C4 and C1q on aggregated IgG in ELISA assays. We confirmed these data by showing that trimodulin reduced the detection of the opsonins C3b and C4b on aggregated IgG concentration-dependently and that trimodulin was more efficient than IVIg (Figure 2A,B). However, the result of C3b/C4b disposition/displacement into the fluid phase could not be confirmed so far (Figure 2C), if we used Ig concentration ranges that are reached in patients (Table 1). Further studies are required to investigate, if trimodulin rather inhibits the C3 amplification loop or MAC lysis pathway without preventing opsonophagocytosis by displacing C3b/C4b. This can be explained by the fact that during early infection, the patient is dependent on pathogen lysis via the alternative pathway (Table 2, situation A), which is enhanced by the amplification loop. As a first immune response, IgM is generated to support opsonophagocytosis (like in Figure 1), which would then allow the amplification loop and MAC pathway to be turned-off and prevent excessive CDC lysis (like in Figure 3, Table 2, situation B). This reasoning would be in agreement with Lutz et al. [61]. At the late stage, where the infection is resolved, IgA and IgG antibodies take over efficient clearance of the pathogens by phagocytosis, whereas IgM concentration is reduced (Table 2, situation C).

The inhibition of CDC by trimodulin, but not by IVIg (Figure 3), most likely occurs at the C3b and C4b step in the cascade and prevents interactions of these opsonins with other factors, e.g., preventing the assembly of C3- and/or C5 convertases. Walpen et al. concluded that IgM preparations in contrast to IVIg are a specific inhibitor of the CP. Thereby, the AP is not inhibited and the lectin pathway only to a lesser, not clinically-relevant extent by IgM preparations. This assures that at least these complement pathways remain fully active to support the anti-pathogenic activity of complement (Table 2, situation E). The IgA molecules in trimodulin (not present in IVIg) support the alternative and lectin pathway in patients treated with trimodulin, but also phagocytosis. The inhibition of CDC by trimodulin is further supported by a previous study. Here, IgM-enriched preparations (Pentaglobin and IVIgM with 70% IgM) prevented the xenogeneic complement activation and CDC-lysis of pig cells by human serum more efficiently compared to IVIg. This mode of action could be translated to in-vivo showing a reduced release of anaphylatoxins as well as a reduced release of pathogenic (toxic, reactive) contents, related to inflammatory processes [23]. Additionally, anaphylatoxins generated by the three different complement pathways, are most likely reduced by scavenging via IgM and/or IgG molecules in trimodulin (Figure 4, [33]). 

In severe infections (Table 2, situation D), the immune system may be overwhelmed with high pathogenic load, their extensive destruction and with large amounts of metabolic products. If pathogens are not sufficiently cleared, inflammation is triggered by an overloaded and overstrained immune system, e.g., via over-activation of the toll-like receptors on the innate immune cells causing a cytokine storm. Furthermore, the IgM and IgG concentration is low in serum, e.g., due to extended consumption or lymphopenia. It is known that patients with sCAP, sepsis or septic shock often exhibit transient antibody depletion (mostly IgM and IgG) and that this condition contributes to disease severity as well as mortality [43]. This deficiency possibly prevents the balance to shift to the anti-inflammatory mode of action. This induces lysis via the alternative and MBL pathways leading to the generation of powerful immune effectors C5a and C3a, inducing chemotaxis as well as activation of more effector cells (e.g., leukocytes or macrophages), which in turn releases more inflammatory mediators like cytokines, lysosomal proteases and reactive oxygen species (ROS) [6,7]. 

Therefore, we assume that patients who show reduced Ig levels during severe infections may benefit from supplementation therapies with Ig preparations like trimodulin to, among other reasons, restore the balance in complement activity, turning off excessive lysis and supporting phagocytosis and opsonophagocytosis activities (Table 2, situation E). Indeed, the clinical phase II trial with trimodulin (CIGMA trial) confirmed that in the patients with sCAP, the IgM levels were significantly reduced and that those patients in particular benefited from trimodulin treatment as shown by a reduced 28-day mortality rate compared to the placebo group [44]. Importantly, the CIGMA trial revealed a beneficial outcome for sCAP patients particularly in those with low IgM and with an elevated inflammation status [44,49]. Whether this is related, at least partially, to restoring the complement balance and reducing complement over-activation and complement-induced hyperinflammation (e.g., C5a release), remains to be investigated in more detail. These findings also support the anti-inflammatory functions of trimodulin and are in accordance with the results generated in this study. In an additional analysis of this data, the overall reduction in inflammation was shown in the trimodulin group (in preparation). 

Several studies have shown that inhibition of the complement system is of clinical importance not only in infections, but also in, for example, autoimmune diseases [36,38,39]. In these diseases, the complement balance is disturbed, insufficiently controlled or the checkpoints of the complement system are circumvented. Multiple therapeutic agents that target the complement system are currently investigated in various autoimmune and inflammatory disorders: (i) Regarding autoimmune diseases like rheumatoid arthritis, systemic lupus erythematosus, vasculitides, Sjögren’s syndrome, and systemic sclerosis evidence revealed that dysregulated complement activation is involved in the pathogenesis, particularly C3, C5aR, CR2, and MAC. Therapeutic strategies include the inhibition of complement activation and complement receptors or membrane attack complex [62,63,64]. Data from preclinical studies and initial clinical trials suggest that the modulation of the complement system could constitute a viable strategy [65,66]. Trimodulin might be another therapeutic option with broader complement modulating activity for these patients and may reduce the pathological complement activation observed in the pathogenesis of autoimmune disease. However, no studies with trimodulin in patients with autoimmune disease have been conducted so far. (ii) Concerning infections, Müller-Redetzky et al. (2020) hypothesized that targeting activated complement components can improve the outcome of pneumonia patients. They reported that inhibition of C5a, which is considerably elevated in sCAP, protects against lung and liver injury in mice with pneumonia. Moreover, high amounts of C5a were also found in the serum or plasma of severe COVID-19 cases [67,68]. Currently, the efficacy of different monoclonal antibodies mostly specifically targeting C3 or C5a complement factors in COVID-19 is investigated in clinical trials (e.g., eculizumab or ravulizumab). There is evidence that these patients positively respond to and benefit from the treatment with complement inhibitors [67,69,70,71]. However, in a recent exploratory trial investigating IFX-1 inhibitor targeting C5a, it was remarked that only inhibiting this molecule might not be sufficient to improve the outcome of patients significantly and that a broader inhibition might be required [72,73]. Compared to such targeted therapy, trimodulin provides a broad mechanism by blocking the complement pathway at C3b/C4b and possibly C5a as well. Furthermore, it balances the complement system between activation and inhibition and not only suppresses the complement, which might be more beneficial in a patient cohort treated without first checking their complement activation status. Balancing allows activation, which is important for the clearance of pathogens and it allows inhibition, protecting the patients against hyperinflammation. In the case a severe infection results in a reduced Ig serum level, this balance cannot be restored and a permanent stimulation (e.g., supported by the amplification loop) of the complement lytic pathway takes place. This could ultimately lead to the complete suppression of the immune system (=immune paralysis) and in the inability of the patient to fight the infection. Under these circumstances, as opposed to monoclonal antibodies, trimodulin prevents this sequence of events and restores the balance. 

Experimental limitations of this work are that the molecular mechanisms by which trimodulin interacts with activated complement factors are not yet evaluated in detail (e.g., scavenging vs. masking). CDC only investigated the effect of trimodulin on the CP, but not on the AP. The data regarding binding to C5a and masking of C3b and C4b on an IgG aggregate surface are preliminary and need further confirmation in other test systems. Furthermore, in this work the distinct contributions of the IgA and IgM isotypes in trimodulin were not investigated, as comparisons were only performed with IVIg. Comparison to IVIg has additional limitations as they are prepared from plasma of different donors and may contain different antibody repertoires (e.g., affecting binding to *S. aureus*). Finally, several assay limitations were encountered, caused by the sticky IgM molecule and the very short-lived C5a molecule. Regarding the analysis in patients, complement was only analyzed in plasma of a very small number of patients and only C3 and C4 factors were analyzed so far. Additional data, e.g., on anaphylatoxin levels, CP and AP pathway activity are missing. Furthermore, plasma levels provide no information on local concentrations at the site of inflammation. 

In future studies it would be of interest to characterize the molecular interactions between different complement factors and the three different isotypes and to analyze the effect of trimodulin treatment on different complement factors in vivo as well as in patients. So far, only C3 and C4 levels have been investigated, which warrants analysis of other players like C5b-9 terminal complex, C5a levels and CH50 and AP50 activities in patients in future clinical trials with trimodulin. The combined in vitro and in vivo ana-lyses could give a better understanding of the detailed mode of action by which trimo-dulin balances the complement system and would further support the potential use of trimodulin for the treatment of inflammation (e.g., in sCAP or severe COVID-19). 

In summary, the data generated in this study give substantial insights into the modes of action of an IgM/IgA-enriched Ig preparation. Trimodulin exhibits dual effects regarding the activity of the complement system and the results are in accordance with various studies that also show dual mechanisms of other Ig preparations in activating and inhibiting the complement. It is assumed that trimodulin helps to reduce inflammation caused by an over-activated complement system, but simultaneously ensures the neutralization of harmful pathogens. Moreover, the data showed crucial advantages of using IgM/IgA-enriched preparation compared to a standard IVIg in severe infections. In addition, the study supports the hypothesis that IgM/IgA-enriched Ig preparations can be a potential adjunctive treatment for the therapy of patients, who suffer from severe inflammatory reactions caused by an over-activated complement system.

## Figures and Tables

**Table 1:** Overview of IgM, IgA and IgG concentrations used in trimodulin assays and detected in humans before and after trimodulin treatment.

Condition	Total Dose	Final Concentration		
		IgM (mg/mL)	IgA (mg/mL)	IgG (mg/mL)
In vitro conditions				
In-assay concentrations	<1.0 mg/mL	<0.23	<0.21	<0.56
	1.6 mg/mL	0.37	0.34	0.90
	2.5 mg/mL	0.58	0.53	1.40
	3.2 mg/mL	0.74	0.68	1.79
	5.0 mg/mL	1.15	1.05	2.80
	6.3 mg/mL	1.45	1.32	3.53
	7.5 mg/mL	1.73	1.58	4.20
	10.0 mg/mL	2.30	2.10	5.60
	12.5 mg/mL	2.90	2.63	7.00
In vivo conditions				
Serum concentrations (normal reference range) ^1^	NA	0.4–2.3	0.7–4.0	7.0–16.0
Serum concentrations (in 24 healthy subjects) ^2^	NA	1.1 (0.55)	2.0 (0.59)	10.4 (2.3)
Serum concentrations (after trimodulin dose in 5–6 healthy subjects) ^2^	1x 91.3 mg/kg	1.2 (0.36)	2.1 (0.41)	11.0 (2.7)
	1x 182.6 mg/kg	1.7 (0.40)	2.8 (0.63)	12.0 (1.8)
	1x 273.9 mg/kg	1.7 (0.13)	2.8 (0.98)	11.2 (2.7)
	5x 182.6 mg/kg	2.9 (0.89)	3.6 (0.40) *	14.6 (1.5) *
Serum concentrations (81 patients with sCAP) ^3^	NA	0.8 (0.79)	2.3 (1.14)	6.8 (2.73)
Serum concentrations (after trimodulin dose in 10–11 patients with sCAP) ^4^	Pre-treatment	0.61 (0.4)	2.6 (0.7)	8.8 (2.8)
	2x 182.6 mg/kg	1.4 (0.5)	3.3 (0.9)	9.3 (1.8)
	4x 182.6 mg/kg	1.9 (0.9)	3.9 (1.1)	10.9 (1.0)
	5x 182.6 mg/kg	2.0 (1.0)	4.1 (1.4)	11.5 (2.3)

^1^: Dati et al. [58]; values depict normal human reference range in mg/mL; ^2^: Trimodulin phase I trial; values depict serum concentration after infusion of the indicated dose at the end-of-infusion on day 1 (1x) or after 5 repeated dosages at the end-of-infusion on day 5 (5x) * or day 6; values depict mean (±SD) in mg/mL; ^3^: Trimodulin phase II trial [44]; values depict mean (±SD) in mg/mL; ^4^: Trimodulin phase II trial [49]; values depict mean (±SD) in mg/mL; NA: not applicable; sCAP: severe community-acquired pneumonia.

**Table 2:** Overview of the influence of IgM, IgA, and IgG on the modes of action of the complement system during the different infection status.

Situation	Infection Status	Immune Status	Mode of Action ^1^	Activity Level ^2^	Balance ^3^
A	Early infection +	IgM increasing Low specific IgG, IgA Low phagocyte numbers at site of infection	Alternative/MBL pathway (IgA/P) → lysis	++	++
			CDC via classical pathway → lysis	+	
			Opsonophagocytosis (IgM) → clearance	+	
			Phagocytosis (IgA, IgG) → clearance	-	
B	Mid infection +++	High IgM Specific IgG, IgA increasing Phagocytes increased at site of infection	Alternative/MBL pathway (IgA/P) →lysis	++	++
			CDC via classical pathway → lysis	-	
			Opsonophagocytosis (IgM) → clearance	+++	
			Phagocytosis (IgA, IgG) → clearance	+	
C	Late infection ++	IgM decreasing Specific IgG, IgA high High number of phagocytes at site of infection	Alternative/MBL pathway (IgA/P) → lysis	+	++
			CDC via classical pathway → lysis	+	
			Opsonophagocytosis (IgM) → clearance	+	
			Phagocytosis (IgA, IgG) → clearance	+++	
D	Severe infection ++++	Low IgM, IgG Specific IgA high Phagocytes impaired at site of infection due to C5a	Alternative/MBL pathway (IgA/P) → lysis	++++	++++
			CDC via classical pathway → lysis	+	
			Opsonophagocytosis (IgM) → clearance	+	
			Phagocytosis (IgA, IgG) → clearance	+	
E	Severe infection + trimodulin ++++	High IgM, IgG Specific IgA high High number of phagocytes at site of infection	Alternative/MBL pathway (IgA/P) → lysis	++	++++
			CDC via classical pathway → lysis	-	
			Opsonophagocytosis (IgM) → clearance	+++	
			Phagocytosis (IgA, IgG) → clearance	+++	

^1^: Mode of action: the alternative pathway includes activation by IgA and pathogens (P) as well as the complement amplification loop resulting in CDC lysis. The classical pathway includes initiation of phagocytosis, opsonophagocytosis and CDC lysis. IgM is expected to attenuate CDC lysis via the classical pathway and possibly the amplification loop; ^2^: Activity level: postulated activity of the different complement pathways inducing either lysis or clearance via phagocytosis; ^3^: Balance: sum of the positive (green, non-inflammatory) and negative (red, inflammatory) immune responses; Color code for activity level and balance: Red: lytic pathway causing cell lysis and inflammation; green: clearance pathway preventing inflammation.

## Data Availability

Data supporting this study can be found in the Ph.D. thesis with the title “Functional characterization of a new IgM- and IgA-enriched immunoglobulin preparation”, which was submitted by C.S. at the Technical University of Darmstadt, Germany (https://doi.org/10.25534/tuprints-00015398), accessd on 17 December 2020.
